# The Risk of Thromboembolism in Patients with Muscle Invasive Bladder Cancer before and after Cystectomy Depending on Blood Group and Neoadjuvant Chemotherapy—A Multicentre Retrospective Cohort Study

**DOI:** 10.3390/jpm13091355

**Published:** 2023-09-04

**Authors:** Emma Schulz Hägersten, Kristoffer Ottosson, Sofia Pelander, Markus Johansson, Ylva Huge, Firas Aljabery, Farhood Alamdari, Johan Svensson, Johan Styrke, Amir Sherif

**Affiliations:** 1 Department of Surgical and Perioperative Sciences, Urology and Andrology, Umea University, 907 36 Umea, Sweden; emma.hagersten@hotmail.com (E.S.H.); krille_ottosson@hotmail.com (K.O.); markus.johansson@rvn.se (M.J.); johan.styrke@umu.se (J.S.); 2 Department of Clinical and Experimental Medicine, Division of Urology, Linkoping University, 581 83 Linkoping, Sweden; sofia.pelander@hotmail.com (S.P.); ylva.huge@regionostergotland.se (Y.H.); firas.abdul-sattar.aljabery@regionostergotland.se (F.A.); 3 Department of Surgery, Urology Section, Sundsvall-Harnosand Hospital, 856 43 Sundsvall, Sweden; 4 Department of Urology, Vastmanland Hospital, 721 89 Vasteras, Sweden; farhood.alamdari@regionvastmanland.se; 5 Department of Statistics, Umea School of Business, Economics and Statistics, Umea University, 907 36 Umea, Sweden; johan.svensson@umu.se

**Keywords:** ABO blood group system, complications, cystectomy, neoadjuvant therapy, thromboembolism, urinary bladder neoplasms

## Abstract

Purpose: Previous studies have indicated that patients with muscle-invasive bladder cancer with non-O blood types have an increased risk of experiencing thromboembolic events (TEEs). This is finding is in relation to neoadjuvant-chemotherapy (NAC)-naïve patients. Aim: to establish the risk of TEEs and any association with blood types among NAC patients as well as NAC-naïve patients. Methods: Cystectomized patients at four centres treated from 2009 to 2018 (*n* = 244) were analysed. The quantities of patients corresponding to each blood group were as follows: A—108 (44%); O—99 (41%); B—30 (12%); and AB—7 (3%). NAC patients (*n* = 167) and NAC-naïve NAC-eligible patients (*n* = 77) were assessed. In total, 54 women (22%) and 190 men (78%), with a median age of 69 years, were included in the study. The occurrence of any type of TEE from six months pre-cystectomy to 12–24 months after was analysed using logistic regression adjusted for NAC and confounders. Results: Sixty-six TEEs were detected in 21% of the patients (*n* = 52). Pulmonary embolus (*n* = 33) and deep venous thrombosis (*n* = 11) were the most common forms. No significant differences between blood types were found in the analysis, although B blood type had a nearly significant increased crude risk compared with O blood type, for which there was an OR of 2.48 (95% CI 0.98–6.36). Adjustment for NAC and covariates weakened the OR, which plummeted to 1.98 (95% CI 0.71–5.51). Conclusions: No significant associations were found between blood types and TEE occurrences in this cohort including both NAC and NAC-naïve NAC-eligible patients.

## 1. Introduction

The gold-standard treatment for medically fit patients with muscle-invasive bladder cancer (MIBC) is platinum-based neoadjuvant combination chemotherapy (NAC) followed by radical cystectomy (RC) [1,2,3]. Trials investigating the effect of NAC on MIBC started in the 1980s [4], and NAC was clinically introduced in the early 2000s. Randomised controlled trials have shown a significant overall survival (OS) benefit of 5–8% in five years compared to the use of RC alone [5,6]. Additionally, much higher survival benefits have been described for complete responders with fully down-staged tumours (pT0N0M0), with an absolute risk reduction for death (ARR) of 31% at five years compared with controls. A complete response to NAC is also considered to be a surrogate marker for improved OS [7]. 

Patients undergoing NAC followed by RC have an increased risk of experiencing thromboembolic events (TEEs) compared to patients undergoing RC alone [8,9,10,11,12]. Zareba et al. followed 202 patients and detected a significantly higher risk of TEEs for patients undergoing NAC compared to RC alone, with an incidence of 19.1% versus 5.6% [12]. In a study by Duivenvoorden et al., which included 761 patients undergoing NAC, a 13.8% incidence of TEEs was detected [8]. In a study involving a smaller cohort, namely, 67 NAC patients compared to 59 well-matched NAC-naïve NAC-eligible patients, we showed that the incidence of TEEs pre-cystectomy in the latter group was only 10% (2/20). Half of the TEEs in the NAC patients were found pre-cystectomy, whereof 11/12 were found during actual NAC-therapy. In 64% of the pre-RC TEEs of the NAC patients, there was an anatomical connection to the placement of a central venous access [9]. Other risk factors for TEEs include the malignancy itself and immobilization [13,14]. Studies have also shown that the occurrence of TEEs is associated with decreased long-term survival [15]. The use of prolonged medical prophylaxis has been suggested to be important for reducing the occurrence of TEEs [16].

Wang et al. were the first to assess the relationship between blood types and the risk of venous thrombotic events (VTE) post-RC, showing a nearly twofold greater risk for patients with non-O blood types (A, B, and AB) [17]. Similar results were later found by Bhanvadia et al. [18] as well as in a meta-analysis by Urabe et al. of over 22,000 patients, of which most were post-operative urological patients, after varying types of surgery, most of which were post-radical prostatectomies. The pooled odds ratio for thrombosis in the full cohort of the cited study was 1.73 (95% CI 1.44–2.10) for patients with non-O blood types [19]. Hypothetically, non-O blood types could promote thrombosis due to a higher rate of circulating von Willebrand Factor [20,21], and a non-O blood type could be a non-modifiable risk factor for TEE in NAC patients, but there are only few data for this group. 

The primary aim of this study was to establish the risk of TEEs and its association with blood types for a group of patients including both NAC and NAC-naïve NAC-eligible patients. This study’s secondary aims included an investigation of a possible association of TEEs with the central venous access, as previously suggested by our group [9].

## 2. Materials and Methods

**Figure 1:**
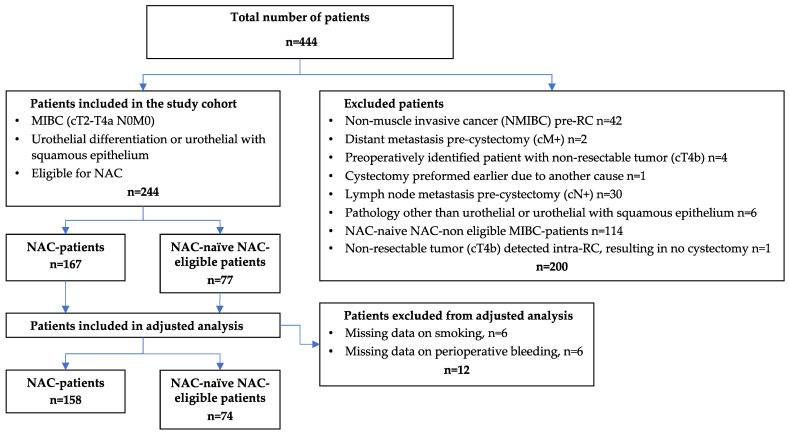
Flowchart of 444 patients with muscle-invasive bladder cancer treated with radical cystectomy divided into NAC patients and NAC-naïve NAC-eligible patients. NAC, neoadjuvant chemotherapy; MIBC, muscle-invasive bladder cancer; NMIBC, non-MIBC; TEE, thromboembolic event.

The study population included all male and female MIBC patients who underwent radical cystectomy from 2009 to 2018 at four Swedish hospitals, namely, Umeå, Linköping, Västerås, and Sundsvall. Both patients with NAC and NAC-naïve NAC-eligible patients were included. An already-existing database was expanded [22], and new variables were added. This resulted in a primary study population of 444 patients. Patients with a staging other than MIBC (cT2-T4N0M0) of urothelial differentiation were excluded along with patients who were not eligible for platinum-based NAC. The presented flowchart outlines the exclusion process, ultimately generating two groups, namely, NAC patients (*n* = 167) and NAC-naïve NAC-eligible patients (*n* = 77), altogether consisting of 244 patients (Figure 1). The NAC-naïve NAC-eligible patients were mainly found in the earlier years since NAC, as a clinical best practice, gradually evolved over time. Important patient characteristics for NAC-eligibility were age ≤ 75 years, kidney function with a glomerular filtration rate (GFR) > 50, and a Charlson Age Comorbidity Index (CACI) of ≤6. Additionally, recommendations from a multidisciplinary team (MDT) were primarily used if MDT meetings had been performed. For VTE prophylaxis pre- and post-RC, most patients (166/244) received low-molecular-weight heparin (Dalteparin or Tinzaparin), whose administration started one day pre-RC and lasted until four weeks after. There was no standard for utilizing VTE prophylaxis during the chemotherapy period.

Data were collected from individual patient medical records. The definition of TEE included recorded clinical or radiological diagnoses of thrombophlebitis; deep venous thrombosis (DVT); arterial embolism; pulmonary embolism (PE), including findings in a control CT before the last NAC cycle [19]; ischemic stroke/transitory ischemic attack (TIA); and myocardial infarction (MI). The occurrences of TEE were observed in five different time periods, ranging (1) from one year before final TUR-B to twenty-four months post-RC. For analyses, the number of patients that experienced TEEs as well as the total numbers of TEEs were used (some patients had >1 TEE). The cut-off date for all observations, including death, was 31 October 2019. Most patients (91%) were followed for 24 months, while the remaining patients were followed for between 12 and 23 months.

A power calculation was conducted, showing that 197 patients would be needed in the O-blood type group and that each of the comparison groups had an 80% chance of detecting a 10% increased risk of a TEE, with a significance level of 5%, in the non-O-blood-type groups if the O-blood-type group had a 10% risk. IBM SPSS statistics, version 26, was used for statistical analysis [23]. Descriptive statistics and medians with interquartile ranges (IQRs) were used to present patient characteristics. Equality of proportions between groups was tested using Fisher’s exact test. The risk of TEE depending on having a different ABO blood group or depending on exposure to NAC was analysed in separate logistic regression models. Patients without complete follow-up data on smoking (*n* = 6) and perioperative bleeding (*n* = 6) were excluded. Adjustments were made for the following cases: 1, NAC in the blood type model and blood type in the NAC model; 2, potential baseline confounders, i.e., sex, age, body mass index (BMI), CACI, and smoking; and 3, potential confounders among perioperative variables, namely, peri-operative bleeding, number of erythrocyte concentrates given because of anaemia during the inpatient time, and length of stay. Data were insufficient for analysing the operating times. The year of cystectomy and treating hospital were closely related to the use of neoadjuvant chemotherapy and were therefore excluded from the analysis. A significance level of 0.05 was used.

## 3. Results

### 3.1. Baseline Data

The cohort comprised 54 women (22%) and 190 men (78%) with a median age of 69 years. In total, 72% of the cohort consisted of current or previous smokers, while the median BMI was 26 and the median CACI was 5. The blood group distribution was as follows: A, 108 (44%); O, 99 (41%); B, 30 (12%); and AB, 7 (3%). Piccline was used by 123 patients (50%), and a Port-a-cath was used by 31 patients (13%). NAC patients had lower Hb levels before the RC than those who did not receive NAC (119 g/L and 139 g/L, respectively), and NAC was more frequently employed at Umeå (81% NAC) than at the other hospitals (55% NAC) and more frequently employed during the latter part of the study period. The median perioperative bleeding levels (750 mL and 1300 mL, respectively) and operation times (399 and 458 min, respectively) were lower in the NAC group compared with the others, and the NAC group had more beneficial pT- and pN-staging values after the RC (Table 1). MVAC-HD/MVAC was the most-used chemotherapy (80.8%), and most NAC patients (64.1%) received three cycles of treatment (Table 2).

### 3.2. Occurrence of TEEs

Throughout the entire observation period, 52 patients (21%) were diagnosed with a TEE, 36 (22%) of which were NAC patients and 16 (21%) of which were NAC-naïve NAC eligible patients. Twelve patients (5%) experienced two TEEs, and one patient had a total of three TEEs. All in all, 66 TEEs were detected during the entire observation period, corresponding to 46 TEEs for the NAC patients and 20 TEEs for the NAC-naïve NAC-eligible patients, respectively (Table 2). The most common type of TEE found was pulmonary embolus, corresponding to 33 cases (50% of the TEEs), and deep venous thrombosis, corresponding to 11 cases (17% of TEE). Nine TEEs were detected in conjunction with CVA. The distribution of TEEs before and after RC showed that the proportion of events before RC was higher in the NAC group, and vice versa among the NAC-naïve NAC-eligible patients (Table 3). 

### 3.3. Association between Blood Types and the Risk of Acquiring a TEE

The distribution of blood types in the patients that did or did not experience a TEE depending on the NAC group is displayed in Table 4. The O blood type was found in 81 (42%) and 18 (35%) of the patients that did not TEE and did experience a TEE, respectively. The corresponding figures for blood type B are 19 (10%) and 11 (21%), respectively. In the logistic regression model, no significant differences were found between O blood type (reference) vs. A, B, and AB blood types, even though B blood showed higher odds ratios (crude OR 2.48, 95% CI 0.98–6.36; adjusted OR 1.98, 95% CI 0.71–5.51) than the other groups (Table 5).

### 3.4. The Risks for Thromboembolic Events in NAC Patients and NAC-Naïve NAC-Eligible Patients

After examining the whole twelve-month period spanning the time before final TUR-B to 24 months after RC, it was determined that there were no significant differences between the crude odds ratios (OR) or the adjusted ORs of TEEs in the NAC-naïve NAC-eligible patients versus the NAC patients (Table 6).

### 3.5. Central Venous Access (CVA) and TEE Incidence

Upon examining the patients that experienced a TEE compared to those that did not experience a TEE, it is evident that a Piccline was used for 30 (60%) members of the former group and 92 (48%) of the latter. A port-a-cath was used for 3 (6%) of the patients that experienced a TEE and 28 (15%) of the patients that did not. No significant differences were found when the occurrence of TEEs depending on port-a-cath or piccline use was analysed with logistic regression, although port-a-caths generally had lower odds ratios than picclines in comparison with no CVA (reference) (crude OR 0.47, 95% CI 0.13–1.73) vs. 1.35, 95% CI 0.69–2.66; adjusted OR 0.62 95% CI 0.14–2.8 vs. 2.03, 95% CI 0.63–6.50) (Table 7). 

TEEs occurring in direct anatomical connection with the establishment of a CVA, as established via radiology, were counted as DVTs and seen in 8/9 TEE incidences in the chemotherapy period of the NAC patients. At the county hospital of Västerås, a Port-a-cath instead of a PICC-line was used for actual NAC administration. Three TEEs occurred in Västerås (amounting to an overall TEE incidence of 8%), two of which occurred in the NAC cohort, and all three only occurred in the early post-RC period, while none occurred in the chemotherapy period. 

## 4. Discussion

Neoadjuvant chemotherapy (NAC) and radical cystectomy (RC) in the treatment of MIBC are known to carry a high risk of the occurrence of a TEE, which can be associated with worse long-term survival [15]. Since NAC was introduced, however, the treatment effects have been predominately positive regarding prognosis and OS [5,6], especially in complete responders [7]. RC in the treatment of urinary bladder cancer has also been identified to carry the highest risk of venous thromboembolism in urologic cancer surgery [24].

Firstly, our analyses showed a TEE incidence of 22% in NAC patients and a significantly increased risk of precystectomy during the period of chemotherapy. These findings can be partly explained by the establishment of a CVA during the actual administration of chemotherapy [9]. Also, the use of CT to control for NAC responses increased the chances of finding pre-RC TEEs in the NAC group.

The main purpose of this study was to evaluate any possible associations between blood group and risk of TEE among MIBC patients that did or did not receive NAC before RC. Wang et al. were the first to suggest that a non-O blood type was a non-modifiable risk factor for thromboembolism for patients undergoing RC [17], which was later confirmed in other studies. Bhanvadia et al. further reported that a non-O blood type is an independent, non-modifiable risk factor for postoperative VTE after RC that is associated with a nearly twofold increased risk. Yet, the study also showed that ABO blood type did not have any significant impact on OS [18]. In urologic cancer surgery, a prostatectomy is another frequently performed intervention that also has a risk of causing TEEs. Tollefsson et al. showed, in a study of over 18,000 patients, an increased risk of TEEs in non-O blood types and an increased 30-day mortality for those who suffered a TEE. Yet, the incidence of TEEs is low, amounting to only 1.4% in the cited study [25].

The main hypothesis being brought forward is that an increased risk of TEE is associated with the amount of circulating von Willebrand Factor (vWF) in the blood [18]. Non-O blood has more circulating vWF, thereby promoting thrombosis, due to vWF serving as a carrier protein for coagulation Factor VIII [21,26,27]. The level of vWF clearance is higher in patients with O blood type; hence, the level of vWF is about 25% lower in the blood of these patients. This may be explained by the glycosyltransferases that determine the non-O blood types (A and B). The glycosyltransferases are involved in the glycosylation process of vWF, in which it becomes resistant to proteolysis and thereby has a longer half-life in the blood [28]. While vWF is increased in non-O blood and acts as a carrier protein, it promotes thrombosis, for example, in association with vascular damage, which might strengthen the hypothesis that the CVA during NAC administration might constitute an important risk factor for thrombosis [13]. Still, our analysis could not show any significant associations between ABO blood types and TEE incidence, and we did not find support for the potential use of blood type for the prediction of TEE risk among NAC-treated MIBC patients. However, a nearly significant increased risk was seen in the B blood type group compared to the O blood type group; in a larger cohort, it might have been significant. Based on the results of the present study and previous studies in the field, future prospective studies could aim to evaluate individualized TEE prophylaxis based on information on blood types. It would be of importance to closely monitor side effects, for example, the occurrence of post-operative bleeding, if higher doses of pharmacological prophylaxis were to be given. Apart from blood types, Corona et al. has suggested an individual-based approach to TEE prophylaxis based on findings of insufficient levels of enoxaparin in patients with a high body mass index [29].

We have previously shown a possible association between anatomical CVA placement and risk of experiencing a TEE [9]. In the present enlarged cohort, we detected 8/9 TEE incidences in the chemotherapy period, all due to CVAs, confirming our previous findings. Interestingly, at the county hospital of Västerås, where the department only used a Port-a-cath (not Piccline) for chemo-administration, there were no incidences of TEEs during NAC-therapy. Yet, a larger cohort would be needed for a more robust analysis of possible negative effects related to different kinds of devices for CVA. The results of a recently published multicentre randomized prospective trial comparing different CVAs (Hickman, Ports, and PICCLINE) with respect to adults aged ≥18 years receiving systemic anticancer treatment via a central vein (≥12 weeks) for solid or haematological malignancy were evaluated. The comparison of Ports and Picclines showed a significant reduction in the overall complication rate of approximately 50% with Ports. This difference was largely explained by a reduction in both mechanical and thrombotic complications when using Ports. The risk of a patient having venous thrombosis was around five times higher with a Piccline than with a Port (2% vs. 11%). Further, pulmonary embolus was rare but more common in the Piccline group [30]. The trial did not include patients with MIBC undergoing NAC, yet the results are of interest for the discussion pertaining to our target population.

There are some limitations of our study; for example, it was retrospective in nature and had a relatively small study population that was slightly underpowered according to the power calculation. The occurrence of TEEs was not evaluated clinically in the same way for all patients; for example, the NAC patients more often had CTs conducted during the pre-operative phase, thus possibly leading to a higher detection rate of TEEs. Patient data were collected from individual medical records, wherein documentation was scarce or missing in a few instances. At NUS hospital, a common obstacle was that patients from other counties in the Northern health region, who underwent surgery in Umeå, had their follow-up routines performed at their local hospitals post-cystectomy. Therefore, the possibility of retrieving postoperative imaging data was often limited, making it plausible that some TEEs might have been lost. Both the studies conducted by Wang et al. and Bhanvadia et al. had relatively large study populations of 2076 patients and 1341 patients, respectively [17,18]. Yet, a major strength of our study was the inclusion of well-matched sub-cohorts, which would presumably have allowed us to adjust for numerous variables if significant results would have been found. A larger study population would be preferable to further investigate if non-O blood type is associated with TEEs suffered by patients undergoing NAC and RC.

## 5. Conclusions

There were no significant associations between ABO-blood types and TEE incidence for MIBC patients who did or did not receive NAC, although B blood type had a twofold higher non-significant risk of TEEs compared with O blood type in the present study. NAC treatment for MIBC involves a high risk of TEE in the period of the actual administration of chemotherapy. The increased risk of NAC-associated TEE seems to be mainly caused by the CVA during chemo-administration, with a suggested exception for patients with Port-a-caths.

## Figures and Tables

**Table 1:** Patient characteristics of NAC-naïve NAC-eligible patients and NAC patients.

Demographic Variable	Subgroup	NAC-naïve NAC-Eligible Patients *n* = 77	NAC-Patients *n* = 167	Total *n* = 244
Sex n (%)	Male	56 (73)	134 (80)	190 (78)
	Female	21 (27)	33 (20)	54 (22)
Age, median (IQR)		68 (9)	69 (8)	69 (9)
CACI, median (IQR)		5 (2)	5 (2)	5 (2)
BMI, median (IQR)		25.2 (5)	25.6 (5)	25.5 (5)
Smoking n (%)	Non-smoker	18 (23)	45 (27)	63 (26)
	Past smoker	35 (45)	79 (47)	114 (47)
	Current smoker	21 (27)	40 (24)	61 (25)
	Missing	3 (4)	3 (2)	6 (2)
Hb pre-RC (g/L) median (IQR)		139 (20)	119 (19)	123 (23)
	Missing n (%)	2 (3)	8 (5)	10 (4)
Year of cystectomy n (%)	2009–2010	39 (51)	19 (11)	58 (24)
	2011–2012	26 (34)	30 (18)	56 (23)
	2013–2014	9 (12)	43 (26)	52 (21)
	2015–2016	1 (1)	44 (26)	45 (18)
	2017–2018	2 (2)	31 (19)	33 (14)
Cystectomy centre n (%)	Umeå	24 (31)	103 (62)	127 (52)
	Sundsvall	12 (16)	30 (18)	42 (17)
	Västerås	10 (13)	30 (18)	40 (16)
	Linköping	31 (40)	4 (2)	35 (14)
Blood group n (%)	A	32 (42)	76 (46)	108 (44)
	B	13 (17)	17 (10)	30 (12)
	AB	1 (1)	6 (4)	7 (3)
	O	31 (40)	68 (41)	99 (41)
Central venous access n (%)	No CVA	69 (90)	20 (12)	89 (37)
	Port-a-cath	5 (7)	26 (16)	31 (13)
	Piccline	2 (3)	121 (73)	123 (50)
	Port-a-cath and piccline	1 (1)	0 (0)	1 (0)
Type of NAC treatment n (%)	MVAC-HD/MVAC		135 (81)	
	MVEC-HD/MVEC		12 (7)	
	Carboplatin Gemzar		8 (5)	
	Other chemotherapy		12 (7)	
Number of NAC cycles n (%)	One cycle		14 (8)	
	Two cycles		21 (13)	
	Three cycles		107 (64)	
	Four cycles		24 (14)	
	Six cycles		1 (1)	
Total operation time (min), median (IQR)		435 (186)	400 (157)	406.5 (162)
	Missing n (%)	23 (30)	81 (49)	104 (43)
Perioperative bleeding (mL), median (IQR)		1300 (1250)	750 (700)	825 (900)
	Missing n (%)	0 (0)	6 (4)	6 (3)
cT-stage n (%)	T2	50 (65)	94 (56)	144 (59)
	T3	27 (35)	61 (37)	88 (36)
	T4a	0 (0)	12 (7)	12 (5)
pT-stage n (%)	T0	12 (16)	58 (35)	70 (29)
	Ta, Tis, T1	5 (7)	27 (16)	32 (13)
	T2	19 (25)	39 (23)	58 (24)
	T3	31 (40)	29 (17)	60 (25)
	T4a	8 (10)	8 (5)	16 (7)
	T4b	2 (3)	6 (4)	8 (3)
pN-stage n (%)	N0	54 (70)	129 (77)	183 (75)
	N1	7 (9)	13 (8)	20 (8)
	N2	12 (16)	12 (7)	24 (10)
	N3	0 (0)	0 (0)	0 (0)
	Missing	4 (5)	13 (8)	17 (7)
pM stage n (%)	M0	76 (99)	167 (100)	243 (100)
	M1	1 (1)	0 (0)	1 (0)
Concomitant prostate cancer n (% of male)	Yes	16 (29)	39 (29)	55 (29)
	No	40 (71)	91 (68)	131 (69)
	Missing	0 (0)	4 (3)	4 (2)
Admission time for RC (days), median (IQR)		16 (3)	14 (6)	15 (5)
Erythrocyte units during RC admission, median (IQR)		2 (4)	2 (4)	2 (4)
	Missing n (%)	1 (1)	1 (1)	2 (1)
Follow-up time n (%)	12–23 months	1 (1)	21 (13)	22 (9)
	24 months	76 (99)	146 (87)	222 (91)

NAC, neoadjuvant chemotherapy; IQR, interquartile range; CACI, Charlson age comorbidity index; BMI, body mass index; CVA, central venous access; RC, radical cystectomy; MVAC/MVEC: M, methotraxate; V, vinblastine; A, adriamycin; E, epirubicin; C, cisplatin; HD, high dose.

**Table 2:** Distribution of 66 thromboembolic events among the 52 patients.

Type of TEE	The NAC-Naïve–NAC-Eligible Patients’ TEEs *n* = 20	The NAC Patients’ TEEs *n* = 46	Total Number of TEEs *n* = 66
Thrombophlebitis, n (%)	0 (0)	1 (2)	1 (2)
Deep venous thrombosis, n (%)	4 (20)	7 (15)	11 (17)
Pulmonary embolus, n (%)	9 (45)	24 (52)	33 (50)
From venous access, n (%)	1 (5)	8 (17)	9 (13)
Stroke/TIA, n (%)	3 (15)	2 (4)	5 (8)
Myocardial infarct/Angina pectoris, n (%)	3 (15)	4 (9)	7 (11)

TEE, thromboembolic event; NAC, neoadjuvant chemotherapy; TIA, transitory ischemic attack.

**Table 3:** Time of diagnosis of thromboembolic events among the patients in the NAC group and the group of NAC-naïve NAC-eligible patients.

NAC	Blood Group	No TEE (*n* = 192), n (%)	TEE (*n* = 52), n (%)	Total (*n* = 244)
NAC-naïve NAC-eligible patients	A	26 (43)	6 (38)	32 (42)
	B	9 (15)	4 (25)	13 (17)
	AB	1 (2)	0 (0)	1 (1)
	O	25 (41)	6 (38)	31 (40)
	Total	61 (100)	16 (100)	77 (100)
NAC patients	A	61 (47)	15 (42)	76 (46)
	B	10 (8)	7 (19)	17 (10)
	AB	4 (3)	2 (6)	6 (4)
	O	56 (43)	12 (33)	68 (41)
	Total	131 (100)	36 (100)	167 (100)

TEE, thromboembolic event; NAC, neoadjuvant chemotherapy; RC, radical cystectomy.

**Table 4:** Distribution of TEEs in the NAC group and the group of NAC-naïve NAC-eligible patients depending on different blood groups.

Time of Diagnosis of TEE	NAC-Naïve NAC-Eligible Patients *n* = 16	NAC Patients *n* = 36	Total *n* = 244
Pre-RC, n (%)	0 (0)	18 (50)	18 (35)
Post-RC, n (%)	15 (94)	16 (44)	31 (60)
Pre- and Post-RC, n (%)	1 (6)	2 (6)	3 (6)

TEE, thromboembolic event; NAC, neoadjuvant chemotherapy.

**Table 5:** Binary logistic regression for thromboembolic events in patients with blood group O vs. groups A, B, and AB.

NAC	Events of TEE (n)	Crude OR (95% CI)	Adjusted OR (95% CI) [Model 1: + Blood Group and Central Venous Access]	Adjusted OR (95% CI) [Model 2: Model 1 + Follow Up Time, Sex, Age, bmi, CACI, and Smoking]	Adjusted OR (95% CI) [Model 3: Model 2 + Per-Operative Bleeding, Number of E-Conc, and Length of Stay]	*p*-Value (Model 3)
NAC-naïve NAC eligible patients (*n* = 74)	16	1 (Ref.)	1 (Ref.)	1 (Ref.)	1 (Ref.)	(Ref.)
NAC patients (*n* = 158)	34	0.99 (0.51–1.94)	0.82 (0.27–2.53)	0.75 (0.23–2.35)	0.65 (0.19–2.26)	0.50

TEE, thromboembolic event; NAC, neoadjuvant chemotherapy; OR, odds ratio; BMI, body mass index; CACI, Charlson age comorbidity index.

**Table 6:** Binary logistic regression for thromboembolic events in NAC-naïve NAC eligible patients and NAC patients.

Blood Groups	Events of TEE (n)	Crude OR (95% CI)	Adjusted OR (95% CI) [Model 1: + NAC and Central Venous Access]	Adjusted OR (95% CI) [Model 2: Model 1 + Sex, Age, bmi, CACI, and Smoking]	Adjusted OR (95% CI) [Model 3: Model 2 + Per-Operative Bleeding, Number of E-Conc, and Length of Stay]	*p*-Value (Model 3)
Blood group O (*n* = 94)	18	1 (Ref.)	1 (Ref.)	1 (Ref.)	1 (Ref.)	(Ref.)
Blood group A (*n* = 104)	20	1.01 (0.50–2.04)	1.06 (0.51–2.18)	1.01 (0.48–2.11)	1.07 (0.51–2.26)	0.86
Blood group B (*n* = 27)	10	2.48 (0.98–6.36)	2.54 (0.98–6.61)	1.97 (0.72–5.38)	1.98 (0.71–5.51)	0.19
Blood group AB (*n* = 7)	2	1.69 (0.30–9.42)	1.58 (0.28–8.95)	1.40 (0.23–8.44)	1.51 (0.24–9.41)	0.66

NAC, neoadjuvant chemotherapy; TEE, thromboembolic event; OR, odds ratio; CI, confidence interval; BMI, body mass index; CACI, Charlson age comorbidity index.

**Table 7:** Binary logistic regression for thromboembolic events among patients with different types of central venous access.

Central Venous Access	Occurrence of TEEs (n)	Crude OR (95% CI)	Adjusted OR (95% CI)[Model 1: + NAC and Blood Group	Adjusted OR (95% CI) [Model 2: Model 1 + Follow Up Time, Sex, Age, bmi, CACI, and Smoking]	Adjusted OR (95% CI) [Model 3: Model 2 + Per-Operative Bleeding, Number of E-Conc, and Length of Stay]	*p*-Value (Model 3)
No CVA (*n* = 86)	17	1 (Ref.)	1 (Ref.)	1 (Ref.)	1 (Ref.)	(Ref.)
Port-a-cath (*n* = 29)	3	0.47 (0.13–1.73)	0.55 (0.13–2.41)	0.61 (0.14–2.69)	0.62 (0.14–2.80)	0.55
Piccline (*n* = 116)	29	1.35 (0.69–2.66)	1.66 (0.55–5.03)	2.06 (0.66–6.45)	2.03 (0.63–6.50)	0.23
Port-a-cath and Piccline (*n* = 1)	1	-	-	-	-	

TEE, thromboembolic event; NAC, neoadjuvant chemotherapy; OR, odds ratio; BMI, body mass index; CACI, Charlson age comorbidity index.

## Data Availability

On reasonable request, the corresponding author can provide all codified data from the clinical database used for this study.
